# Impact of Vitamin B12 Supplementation on Cardiovascular Health in the Silver Star Bamboo Rat, a Species That Feeds Primarily on Bamboo

**DOI:** 10.3390/ani15172526

**Published:** 2025-08-27

**Authors:** Lei Chen, Zhoulong Chen, Yongqi Zhao, Nan Yang, Jingheng Wang, Yanni Zhao, Lijun Luo, Xiuyue Zhang

**Affiliations:** 1 Key Laboratory of Bio-Resources and Eco-Environment, Ministry of Education, College of Life Sciences, Sichuan University, Chengdu 610064, China; geminist_cl@163.com (L.C.); chenzhoulong1010@163.com (Z.C.); 18772837879@163.com (Y.Z.); wangjingheng@stu.scu.edu.cn (J.W.); zhaoyanni_69@163.com (Y.Z.); luolijun19970120@163.com (L.L.); 2 Key Laboratory of Qinghai-Tibetan Plateau Animal Genetic Resource Reservation and Utilization, Sichuan Province and Ministry of Education, Southwest Minzu University, Chengdu 610225, China; yangnan0204@126.com; 3 The Conservation of Endangered Wildlife Key Laboratory of Sichuan Province, College of Life Sciences, Sichuan University, Chengdu 610064, China

**Keywords:** bamboo rat, cardiovascular disease, vitamin B12 supplementation, liver transcriptome, serum biochemical analysis

## Abstract

Specialized herbivores like giant pandas (*Ailuropoda melanoleuca*), red pandas (*Ailurus fulgens*), and bamboo rats, which primarily consume bamboo, are at risk of nutrient deficiencies, particularly vitamin B12 (VB12), potentially leading to cardiovascular diseases. This study explored the effects of VB12 supplementation on cardiovascular health in silver star bamboo rats (*Rhizomys pruinosus*). We first conducted a comprehensive genome annotation of *R. pruinosus*, laying the foundation for in-depth evolutionary studies. Comparative transcriptomic analysis revealed that genes related to cardiovascular disease (e.g., *Sgcb*, *Adcy2*, *Itga1*, *Itgb8*, *Ifng*, and *Gpc1*) were upregulated in the livers of *R. pruinosus* compared to carnivorous and omnivorous rodents, indicating a higher cardiovascular disease risk. After 60 days of VB12 supplementation, liver transcriptome analysis revealed significant improvements in cardiovascular health markers, including reduced cholesterol synthesis and enhanced fatty acid metabolism. Serum biochemical assays indicated that VB12 supplementation led to reduced homocysteine levels, decreased low-density lipoprotein (LDL)-to-high-density lipoprotein (HDL) ratios, and increased the apolipoprotein A-to-apolipoprotein B ratio. These findings suggest that VB12 may mitigate cardiovascular disease risk and could be considered in the dietary management of specialized bamboo-eating species. Our study provides valuable insights into disease prevention strategies for these species with similar dietary habits.

## Simple Summary

This study explores the effects of vitamin B12 supplementation on cardiovascular health in bamboo rats, a species with a diet that lacks this essential nutrient. Our findings show that vitamin B12 supplementation reduces homocysteine levels and improves lipid metabolism, which may help mitigate cardiovascular risks. These results are significant for the conservation of bamboo-eating species like the bamboo rat, and could also inform dietary strategies for other animals with similar diets. The study highlights the potential of vitamin B12 to improve animal health and reduce disease risks in species at risk of nutrient deficiencies.

## 1. Introduction

Studies have indicated significant differences in the incidence and mortality rates of cardiovascular diseases (CVDs) between vegetarians and non-vegetarians [1,2]. A key factor is the absence of vitamin B12 (VB12) in vegetarian diets, which can lead to elevated homocysteine (Hcy) levels [3,4]. Elevated Hcy is associated with endothelial dysfunction and is considered an independent risk factor for CVDs. Higher serum Hcy levels can directly or indirectly inflict damage on vascular endothelial cells, increase endothelin secretion, disrupt the balance between nitric oxide and endothelin, and promote arteriosclerosis. Moreover, Hcy-induced oxidative stress can damage endothelial cells, stimulate vascular smooth muscle cell proliferation, and enhance platelet aggregation and adhesion, thereby fostering thrombosis [5,6]. Homocysteine metabolism predominantly follows two pathways: remethylation and transsulfuration. In the remethylation pathway, Hcy is converted back to methionine via methionine synthase (MS), which requires VB12 (cobalamin) as a cofactor and 5-methyltetrahydrofolate as a methyl donor, facilitated by methylenetetrahydrofolate reductase (MTHFR). VB12 deficiency impairs this pathway, leading to Hcy accumulation. Alternatively, in the transsulfuration pathway, Hcy is irreversibly converted to cystathionine by cystathionine β-synthase (CBS), requiring vitamin B6, and further to cysteine. However, when remethylation is blocked, excess Hcy may overload transsulfuration, exacerbating toxicity [7,8,9]. Hcy toxicity mechanisms include induction of reactive oxygen species (ROS), leading to lipid peroxidation and DNA damage; activation of inflammatory pathways (e.g., NF-κB signaling); and epigenetic alterations, such as reduced histone methylation (e.g., H3K36me3), which may promote atherosclerosis [10,11]. In vegetarian or low-B12 diets, these effects undermine cardiovascular protection despite low cholesterol intake [2]. VB12, which cannot be synthesized endogenously, is primarily sourced from meat, eggs, milk, animal organs, and various fermented soy products [12].

The silver star bamboo rat (*Rhizomys pruinosus*), giant panda (*Ailuropoda melanoleuca*), and red panda (*Ailurus fulgens*) exemplify dietary specialization, with diets predominantly consisting of bamboo [13,14]. Despite bamboo being low in fat and free of cholesterol [15], both giant pandas and red pandas show a predisposition to CVDs, particularly atherosclerosis. Studies indicate that cardiovascular issues are a major mortality factor in adult red pandas [16], with atherosclerosis, congestive heart failure, and myocarditis cited as frequent causes of death in giant pandas [17]. We hypothesize that this susceptibility, akin to that observed in vegetarians, stems from a VB12-deficient bamboo diet, where impaired Hcy metabolism amplifies endothelial damage and oxidative stress. To compensate for this dietary deficiency, both panda species appear to have developed adaptive mechanisms. For instance, the adaptive evolution of the *GIF* and *CYP4F2* genes enhances VB12 absorption, thereby alleviating the cardiovascular consequences of VB12 deficiency [18,19]. Additionally, the gut microbiota of pandas contributes to endogenous VB12 synthesis [20]. Nevertheless, these strategies seem insufficient to fully counteract the high prevalence of CVDs in pandas [16,21]. Reducing the risk of CVDs in pandas and providing better protection remains a critical focus of current research.

Given its bamboo-based diet, *R. pruinosus* serves as a valuable model for investigating the effects of VB12 deficiency [22,23]. Ecologically, this subterranean rodent inhabits bamboo-rich environments in Southeast Asia, feeding primarily on bamboo roots, shoots, and stems, mirroring the lignocellulose-heavy diet of pandas and thus facing similar risks of nutrient deficiencies like VB12 [23]. Physiologically, its gut microbiome undergoes adaptations, such as a shift from Bacteroidaceae to Muribaculaceae dominance, to degrade lignocellulose and potentially synthesize VB12 via microbial pathways, yet these may not fully mitigate deficiency-related CVD risks. As a rodent, it offers practical advantages for laboratory studies, including ease of breeding and sampling compared to larger pandas [24]. In this study, we first annotated the genome of *R. pruinosus* to establish a genomic reference for transcriptomic analysis. We then compared the liver transcriptomes of *R. pruinosus* with those of carnivorous and omnivorous rodents to identify CVD-related gene expression patterns and assess whether bamboo rats, like giant pandas and red pandas, exhibit increased cardiovascular risk. Finally, we supplemented the diet of *R. pruinosus* with VB12 and integrated transcriptomic and serum biochemical analyses to investigate the impact of VB12 on cardiovascular-related gene expression. Through these investigations, we aimed to elucidate the role of VB12 in maintaining cardiovascular health in species with inherently VB12-deficient diets.

## 2. Materials and Methods

### 2.1. Structural and Functional Annotation of the R. pruinosus Genome

The genome assembly of *R. pruinosus* (version RhiPru_1.0) was obtained from the NCBI database. Repetitive elements within the genome were identified using a combined strategy involving both homology-based and de novo predictions, implemented via RepeatMasker and RepeatModeler [25,26]. Following the masking of repetitive sequences, structural gene annotation was performed using an integrative approach that incorporated homology-based, transcriptome-based, and ab initio prediction methods. Protein-coding gene models were inferred based on protein homology with six reference species (*Homo sapiens*, *Mus musculus*, *Rattus norvegicus*, *Mesocricetus auratus*, *Nannospalax galili*, and *Oryctolagus cuniculus*) using GeneWise version 2.4.1 [27]. In parallel, RNA-seq reads were aligned using HISAT2 version 2.2.1 [28] and ab initio gene predictions were conducted with AUGUSTUS v 3.0.8, trained on the transcriptomic data generated in this study [29,30]. The annotation pipeline Geta (https://github.com/chenlianfu/geta, accessed on 2 December 2023) was used to integrate the results from all three approaches to generate a comprehensive, non-redundant set of protein-coding gene models. Functional annotations were further assigned using the eggNOG database [31].

### 2.2. Laboratory Animals and Experimental Groupings

All experimental procedures and animal care practices followed the guidelines approved by the Experimental Animal Management and Use Committee of Sichuan University (license 20190506001).

A total of six *R. pruinosus* individuals, born in the same batch to ensure genetic consistency, were selected and maintained under the same environmental conditions. The sex and developmental stage of all animals are detailed in Supplementary Table S1. Health status was monitored throughout the study to confirm the absence of disease, and all animals were screened to ensure they were in a healthy physiological state at the time of inclusion in the study. The animals weighed approximately 1100–1200 g at the beginning of the experiment. Animals were randomly assigned to two groups (*n* = 3 per group): the VB12 group and the control group (details in Supplementary Table S1). Both groups received the same basal diet, but with different supplementation regimens. The VB12 group was fed basal feed supplemented with VB12, while the control group received the same basal feed without any additional supplements. All animals received the same basal formulated diet consisting (by weight) of rice (30%), rice bran (15%), cornmeal (15%), and bamboo stems (40%), offered at a fixed ration of 60 g per animal per day. The VB12 group received the identical basal diet fortified with VB12 at ~167 µg/kg feed, which corresponds to a target dose of 10 µg VB12 per animal per day based on the 60 g ration (10 µg ÷ 0.06 kg = 166.7 µg/kg). This dose was selected based on prior studies showing effective homocysteine (Hcy) reduction and cardiovascular protection at comparable levels. For instance, in hyperhomocysteinemic rat models, VB12 at 100–150 μg/day (i.m.) attenuated myocardial infarction [32]. While this dose was higher for treating induced deficiency states, our lower oral dose aligned with nutritional supplementation ranges (e.g., 0.4–1 mg/day in humans, scaled to ~5–12.5 μg/day in rodents for safe Hcy reduction without toxicity [4,33,34]). The control group received the same basal diet without VB12. VB12 was mixed into each animal’s daily basal ration immediately before feeding. Since all animals consumed the entire 60 g of the provided basal diet, the actual VB12 exposure in the VB12 group was approximated by basal diet consumption: 10 µg/day.

The 60-day intervention period was selected to capture steady-state physiological responses to vitamin B12 supplementation. In rodents, the red blood cell (RBC) lifespan typically ranges from 42 to 65 days [35,36]. Therefore, the 60-day duration aligns with one full erythrocyte turnover cycle, making it particularly relevant for studying changes in one-carbon metabolism and homocysteine dynamics, both of which are influenced by VB12 status. Previous studies on rodents have shown measurable improvements in homocysteine and lipid metabolism within 4–12 weeks of B12 supplementation, and 8 weeks is a commonly used intervention window in dietary studies [33].

Due to welfare concerns and blood-volume limitations in *R. pruinosus*, baseline blood samples were not collected for this study. The total blood volume of an adult bamboo rat is relatively small, and repeated blood collection could interfere with hematological homeostasis. Instead, we focused on endpoint measures to assess the effects of the intervention on serum biochemical markers. These ethical considerations were adhered to as part of the study design, ensuring minimal impact on animal welfare.

### 2.3. Sample Collection and Detection of Serum Biochemical Indicators

Following the two-month dietary intervention, sample collection was initiated. No significant differences in body weight or growth parameters were observed between the VB12 and control groups. The *R. pruinosus* individuals were euthanized after a 12 h fasting period. Blood samples were collected via cardiac puncture, and liver tissues were simultaneously harvested. Liver samples were immediately frozen in liquid nitrogen and stored at –80 °C to preserve RNA and protein integrity. Blood samples were centrifuged to obtain serum, which was subsequently submitted to Jiangsu Yutong Biotechnology Co., Ltd. (Changzhou, China) for biochemical analysis. The measured serum indicators included homocysteine (Hcy), glucose (GLU), high-density lipoprotein (HDL), low-density lipoprotein (LDL), apolipoprotein A (APO-A), and apolipoprotein B (APO-B). Specifically, APO-A, APO-B, and Hcy were quantified using the company’s enzyme-linked immunosorbent assay (ELISA) kits following manufacturer protocols. GLU was measured via the glucose oxidase method, while HDL and LDL were determined using the two-point endpoint method.

### 2.4. Library Construction and Sequencing of R. pruinosus Samples

Prior to sequencing, liver tissues from all six *R. pruinosus* individuals were dissected and homogenized. Total RNA was extracted using TRIzol reagent (Invitrogen, Carlsbad, CA, USA) and treated with RNase-free DNase to remove genomic DNA contamination. Only samples with RNA integrity numbers (RINs) > 5.8 were selected for subsequent RNA-seq library construction. Libraries were prepared using the NEBNext^®^ Ultra™ RNA library preparation kit from Illumina^®^ (NEB, Ipswich, MA, USA) according to the manufacturer’s protocol, and index codes were added to uniquely identify each sample. Following column purification, library quality was assessed using an Agilent Bioanalyzer 2100 system. Libraries were sequenced on the Illumina NovaSeq 6000 platform (Illumina, San Diego, CA, USA). The resulting sequencing reads have been deposited in the NCBI Sequence Read Archive (BioProject Accession PRJNA1140354).

### 2.5. Quality Control and Alignment of Raw Sequencing Data

Transcriptome sequencing was performed on liver samples from *R. pruinosus*, and additional liver transcriptome data were collected from several carnivorous and omnivorous rodent species, including the Sikkim mouse (*Mus pahari*), house mouse (*Mus musculus*), rat (*Rattus norvegicus*), and golden hamster (*Mesocricetus auratus*). The sex and developmental stage of all the animals used for sequencing are provided in Supplementary Table S1. Quality control of the raw sequencing reads was performed using the NGS QC Toolkit (version 2.3.3) to remove low-quality reads and adapter sequences [37]. The quality of the filtered reads was further assessed using FastQC (version 0.11.9) (http://www.bioinformatics.babraham.ac.uk/projects/fastqc/, accessed on 2 December 2023).

High-quality reads were aligned to the corresponding reference genomes using HISAT2 version 2.2.1 [38]. SAMtools version 1.21 [39] was employed to convert the resulting alignment files into BAM format for further analysis. For *R. pruinosus*, the reference genome (GCA_009823505.1_RhiPru_1.0) and associated annotation were retrieved from GenBank. The reference genome for the Sikkim mouse was obtained from RefSeq (version GCF_900095145.1_PAHARI_EIJ_v1.1), while genome assemblies and annotations for the remaining rodents were acquired from Ensembl release 110. Gene expression quantification was performed using featureCounts from the Subread package version 2.0.6 [40], generating read counts for downstream transcriptomic analyses.

### 2.6. Principal Component Analysis and Spearman Correlation Clustering

To investigate interspecies relationships and clustering patterns among *R. pruinosus* and other non-herbivorous rodents, we first identified one-to-one single-copy orthologous genes across five species. Protein-coding sequences were retrieved from each species’ genome and filtered by applying the following criteria: (i) removal of sequences shorter than 50 amino acids; (ii) exclusion of sequences containing premature stop codons; and (iii) retention of only the longest isoform for each gene. Orthologous genes were identified using OrthoFinder (version 2.3.7) [41], based on BLASTp alignments (E-value cutoff: 1 × 10^−5^) with a reciprocal best hit strategy. A total of 3189 single-copy orthologous genes were identified for further analyses. To account for differences in sequencing depth and gene length across species, expression data were normalized using the GeTMM method [42], which integrates gene length correction with TMM normalization. A gene expression matrix was constructed with genes as rows and samples as columns. After removing genes that were not expressed in any sample of a given species, normalized gene expression values were log2-transformed. Principal component analysis (PCA) was performed using the prcomp function from the R package (version 4.8.3) stats, and results were visualized using ggplot2 [43]. Spearman correlation clustering was conducted using the cor function from stats and visualized with the heatmap.2 function from the gplots package.

### 2.7. Identification of Differentially Expressed Genes (DEGs)

To comprehensively compare gene expression patterns across species, we used the filterByExpr function from the R package edgeR to filter out low-expression genes from the raw expression matrices of each species [44]. This function retains genes with at least 10 read counts in a number of samples equal to the size of the smallest experimental group. To account for potential technical variability, sequencing depth, and gene length differences, normalization was performed using the TMM algorithm within edgeR. Subsequent analyses focused on gene expression differences between the control group of *R. pruinosus* and various non-herbivorous rodents. Genes were considered significantly differentially expressed (DEGs) if they exhibited an absolute log2 fold change (|log2FC|) ≥ 1 and a Benjamini–Hochberg-adjusted *p*-value (FDR) < 0.05.

To explore gene expression differences among dietary intervention groups in *R. pruinosus*, the same pipeline was applied. Low-expression genes were filtered using filterByExpr, and TMM normalization was conducted to correct for technical variability, sequencing depth, and gene length. Gene expression changes between the VB12 group and the control group were assessed. Genes were classified as significantly differentially expressed if they had a Benjamini–Hochberg-adjusted *p*-value (FDR) < 0.05 and an absolute log2 fold change (|log2FC|) ≥ 1.

### 2.8. Enrichment Analysis of Differentially Expressed Genes

Functional enrichment analysis of the identified DEGs was performed to identify enriched Gene Ontology (GO) terms and Kyoto Encyclopedia of Genes and Genomes (KEGG) pathways. For the within-species comparison (VB12 vs. control groups in *R. pruinosus*), we used the enricher function from the clusterProfiler R package (version 4.8.3) [45], with the background set as the annotated protein-coding genes of *R. pruinosus* from our genome annotation (Section 2.1). GO annotations were obtained from EggNOG v5.0 (http://eggnog5.embl.de/#/app/home, accessed on 2 December 2023), and KEGG pathway information was retrieved from the KEGG database (https://www.genome.jp/kegg/pathway.html, accessed on 2 December 2023). Enrichment was considered significant at *p* < 0.05.

For the interspecies comparative analysis (*R. pruinosus* vs. non-herbivorous rodents), enrichment was conducted using KOBAS (version 3.0) [46], with mouse (*Mus musculus*) as the background gene set due to orthologue mapping and more comprehensive annotations available for rodents. KOBAS performed KEGG and GO enrichment with Benjamini–Hochberg correction, and pathways/terms were significant at adjusted *p* < 0.05. Disease associations were inferred from KEGG pathways involving CVD.

### 2.9. Protein–Protein Interaction (PPI) Network Analysis

To identify interaction networks and hub genes among DEGs influenced by VB12 supplementation, PPI analysis was performed using the STRING database (version 12.0) [47], and network analysis and core gene (hub gene) identification were conducted using Cytoscape software (version 3.7.1).

Protein interaction data obtained from the STRING database were filtered using the default parameters (interaction score > 0.4) and exported as interaction relationship files. The PPI network for DEGs was constructed using Cytoscape software [48]. The network analysis tool in Cytoscape was further used to calculate the degree of each gene node. Genes with higher degrees were defined as core hub genes in the network. These core genes are likely to play important regulatory roles in the high-altitude adaptation of musk deer and provide key target information for subsequent experimental validation and functional studies.

### 2.10. RT-qPCR Validation of Key DEGs

To validate key differentially expressed genes from the comparative transcriptomic analysis, real-time quantitative PCR (RT-qPCR) was performed on liver samples from *Rhizomys pruinosus* and non-herbivorous rodents (*Mus musculus*, *Rattus norvegicus*, and *Mesocricetus auratus*). Total RNA was extracted using an M5 Universal RNA Mini Kit (Mei5 Biotechnology, Beijing, China), and cDNA was synthesized with an M5 Sprint qPCR RT Kit (Mei5 Biotechnology, China). qPCR was conducted on a CFX96 real-time PCR detection system using SYBR Premix (Mei5 Biotechnology, China) with gene-specific primers (sequences in Supplementary Table S2). *ACTB* was used as the internal reference gene. Relative expression was calculated via the 2^−ΔΔCt^ method. Validation was successful for *Sgcb*, *Itga1*, *Itgb8*, and *Ifng*. Attempts for *Adcy2* and *Gpc1* failed due to unsuccessful primer design.

## 3. Results

### 3.1. Structural Annotation of the Bamboo Rat Genome

The *R. pruinosus* genome encompasses 3.71 billion base pairs, of which 36.33% are classified as repetitive sequences. We employed a multifaceted gene prediction approach, integrating homology-based, transcriptome-based, and ab initio methodologies, and successfully identified 26,618 genes. A notable RNA-seq read alignment rate of 94% supports the high quality of our transcriptomic data, which further enhanced the accuracy of homology-based gene predictions. The AUGUSTUS training model achieved an overall prediction accuracy of 67.5%, with nucleotide-, exon-, and gene-level sensitivities of 92.4%, 76.9%, and 19.0% and specificities of 76.8%, 74.7%, and 12.1%, respectively. Repetitive sequences are primarily composed of interspersed retroelements, notably long interspersed nuclear elements (LINEs, 9.81%), and short interspersed nuclear elements (SINEs, 13.42%), as well as a diverse array of DNA transposons and other repetitive elements.

### 3.2. Overview of Liver Transcriptomes of R. pruinosus and Non-Herbivorous Rodents

**Figure 1:**
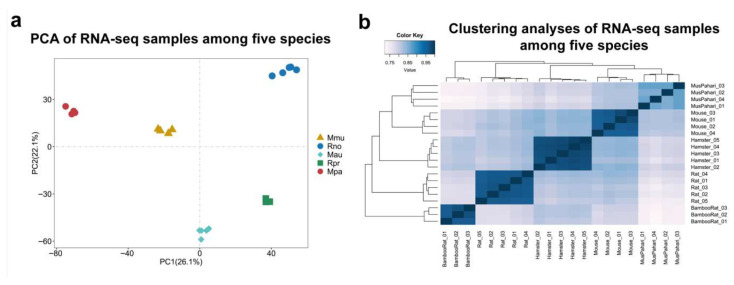
Principal component analysis (PCA) and clustering analyses of all samples among five species. (**a**) PCA of the log-transformed normalized expression levels of all orthologous genes across liver samples from different species. Species are represented by point shapes and colors. (**b**) Clustering analyses of the log-transformed normalized expression levels of all orthologous genes across liver samples from different species. Distance between samples measured by Spearman’s rank correlation coefficient. Abbreviations: Mmu: mouse, Rno: rat, Mau: golden hamster, Rpr: silver star bamboo rat, Mpa: sikkim mouse.

Our analysis revealed alignment rates for liver transcriptomes ranging from 70.08% to 96.40% across *R. pruinosus* and four non-herbivorous rodent species, as shown in Supplementary Table S1. We identified 3189 1:1 single-copy orthologous genes shared among the five species. Principal component analysis (PCA) and Spearman’s correlation distance clustering were used to evaluate the expression profiles of each sample (Figure 1a,b). PCA results showed that liver samples from the same species clustered tightly together, while those from different species clustered separately. Spearman’s correlation distance clustering further revealed distinctions in dietary adaptation: the carnivorous Sikkim mouse initially clustered with the omnivorous species (house mouse, golden hamster, and rat), and was clearly distinct from the bamboo-feeding *R. pruinosus*.

### 3.3. Identification and Enrichment Analysis of DEGs Between R. pruinosus and Non-Herbivorous Rodents

**Figure 2:**
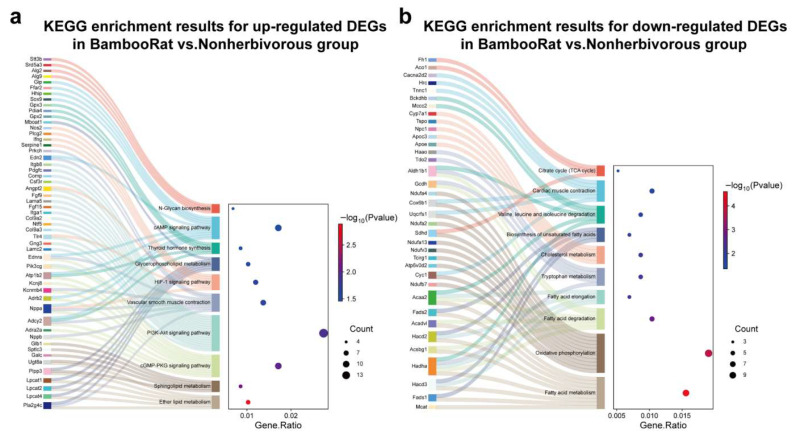
Significantly enriched key KEGG pathways of differentially expressed genes (DEGs) in *R. pruinosus* vs. non-herbivorous group. (**a**) Significantly enriched key KEGG pathways for upregulated DEGs in *R. pruinosus* vs. non-herbivorous group. (**b**) Significantly enriched key KEGG pathways downregulated DEGs in *R. pruinosus* vs. non-herbivorous group. The top 10 key enriched pathways are shown. The *X*-axis of the dot plot indicates the GeneRatio of the KEGG-enriched pathways, and the *Y*-axis indicates the name of the pathway.

A total of 1161 DEGs were identified between *R. pruinosus* and four non-herbivorous rodents, including 584 upregulated and 577 downregulated genes (Supplementary Figure S1). GO and KEGG pathway enrichment analyses were conducted on the identified DEGs using KOBAS (Figure 2; Supplementary Tables S3–S6).

Upregulated genes in *R. pruinosus* were significantly enriched in metabolic pathways related to ether lipid metabolism, sphingolipid metabolism, and glycerophospholipid metabolism (Figure 2a). This suggests that *R. pruinosus* has a stronger capacity to metabolize bioactive lipid and structural lipids than non-herbivorous rodents, possibly reflecting their distinct metabolic demands. In contrast, downregulated genes were significantly enriched in several key nutrient metabolism pathways, including fatty acid metabolism, oxidative phosphorylation, and fatty acid degradation, suggesting a reduced capacity for lipid utilization (Figure 2b). This is consistent with the low-fat content of their bamboo-based diet. Additionally, pathways such as fatty acid elongation, biosynthesis of unsaturated fatty acids, cholesterol metabolism, tryptophan metabolism, degradation of branched-chain amino acids (valine, leucine, and isoleucine), and the tricarboxylic acid (TCA) cycle were also downregulated.

While the most significantly enriched upregulated pathways involved lipid metabolism (e.g., ether lipid metabolism, sphingolipid metabolism, glycerophospholipid metabolism), several upregulated DEGs participated in CVD-related KEGG pathways, such as dilated cardiomyopathy (DCM; KEGG mmu05414), arrhythmogenic right ventricular cardiomyopathy (ARVC; KEGG mmu05412), hypertrophic cardiomyopathy (HCM; KEGG mmu05410), and fluid shear stress and atherosclerosis (KEGG mmu05418), though these were not the top enriched terms (Supplementary Tables S5 and S6). Key genes from these pathways include *Sgcb*, *Adcy2*, *Itga1*, *Itgb8*, *Ifng*, and *Gpc1*, which showed significant upregulation and are linked to cardiovascular dysfunction [49,50,51,52,53,54,55,56,57]. These findings suggest a heightened risk of cardiovascular dysfunction in *R. pruinosus*, potentially associated with their unique dietary habits. To confirm these observations, RT-qPCR validation was performed on selected upregulated CVD-related genes (*Sgcb*, *Itga1*, *Itgb8*, and *Ifng*), showing that the changing trends of their expression levels were consistent with RNA-seq results (Supplementary Figure S3). Validation for *Adcy2* and *Gpc1* was not possible due to primer design failure.

### 3.4. Determination of Serum Biochemical Indicators in Different Dietary Intervention Groups of R. pruinosus

**Figure 3:**
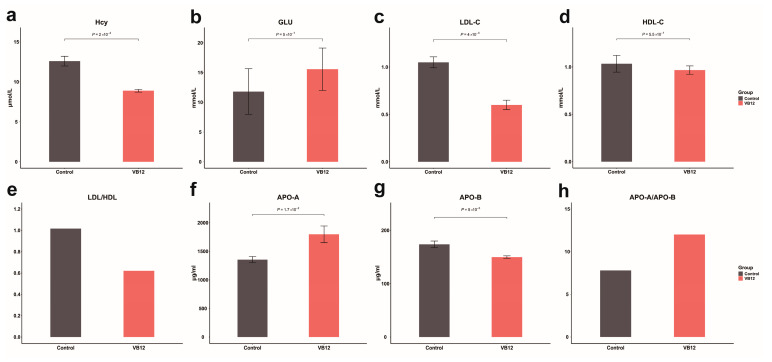
Levels of different serum biochemical indicators in different dietary intervention groups of *R. pruinosus* (*n* = 3 per group). Data are presented as means ± SE. Statistical significance was determined using independent *t*-tests. Note: Ratios (LDL/HDL and APO-A/APO-B) were calculated as means of individual ratios without reported error values, as they were derived metrics. The *X*-axis indicates different dietary intervention groups, and the *Y*-axis indicates the levels of serum biochemical indicators.

We evaluated several key serum biochemical indicators in *R. pruinosus* under different dietary interventions (*n* = 3 per group), such as homocysteine (Hcy), glucose (GLU), high-density lipoprotein (HDL), low-density lipoprotein (LDL), apolipoprotein A (APO-A), and apolipoprotein B (APO-B) (Figure 3).

Statistical significance was assessed using independent *t*-tests. Serum Hcy levels were significantly lower in the VB12 group compared to the control group (*p* < 0.01), indicating that VB12 supplementation effectively reduces Hcy accumulation. This reduction likely contributes to a lower risk of CVDs by improving endothelial function and lowering oxidative stress. Notably, HDL levels did not significantly differ between the two groups (*p* > 0.05), whereas LDL levels were significantly decreased (*p* < 0.01), suggesting a beneficial alteration in lipid metabolism. The decreased LDL/HDL ratio in the VB12-supplemented group further supports the protective role of VB12 in mitigating atherosclerosis risk by maintaining a healthier lipid profile. Similarly, apolipoprotein analysis revealed that APO-A levels were significantly elevated in the VB12 group (*p* < 0.05), while APO-B levels were significantly reduced (*p* < 0.01). The resulting increase in the APO-A/APO-B ratio suggests a favorable shift in the balance between anti-atherogenic and pro-atherogenic lipoproteins. Interestingly, no significant difference in glucose levels was observed between the groups (*p* > 0.05), indicating that VB12 supplementation does not noticeably affect glucose metabolism in *R. pruinosus*. Collectively, these findings demonstrate that VB12 supplementation significantly reduces cardiovascular risk factors by lowering Hcy levels and improving lipid profiles.

### 3.5. Overview of Liver Transcriptomes of Different Dietary Intervention Groups Within R. pruinosus

**Figure 4:**
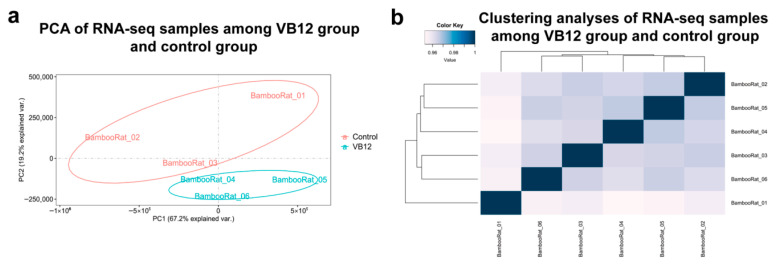
PCA and clustering analyses of the mRNA expressions for VB12 and control group. (**a**) PCA of the log-transformed normalized expression levels of all samples in the VB12 group and control group. Different groups are represented by different colors. (**b**) Clustering analyses of the log-transformed normalized expression levels of all samples in the VB12 group and control group. Distance between samples was measured by Spearman’s rank correlation coefficient.

To explore gene expression variations among dietary intervention groups in *R. pruinosus*, we performed PCA and Spearman correlation distance clustering (Figure 4). The PCA revealed a clear separation between the VB12 group and the control group, indicating that VB12 supplementation influenced overall gene expression profiles. However, the Spearman-based clustering did not exhibit a distinct separation between the two groups, suggesting that the effects of VB12 may be localized to specific pathways or gene clusters rather than causing widespread transcriptomic changes.

### 3.6. Identification and Enrichment Analysis of DEGs Between Different Dietary Intervention Groups Within R. pruinosus

RNA-seq data from the VB12 and control groups were compared to identify DEGs (Supplementary Figure S2). A total of 62 DEGs were identified, including 16 upregulated and 46 downregulated genes.

**Figure 5:**
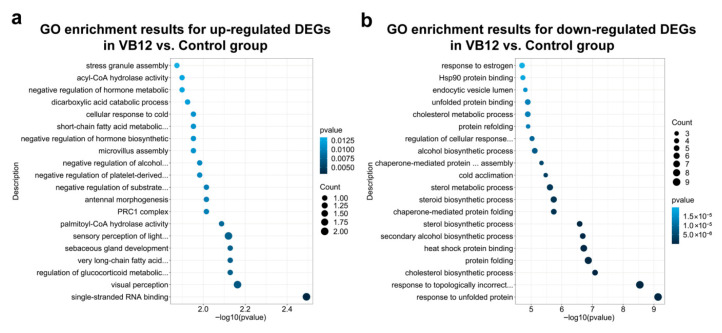
Significantly enriched KEGG pathways of DEGs in VB12 group vs. control group. (**a**) Significantly enriched GO categories for upregulated DEGs in VB12 group vs. control group. (**b**) Significantly enriched GO categories for downregulated DEGs in VB12 group vs. control group. The 20 most significantly enriched items are shown. The *X*-axis indicates the −log10 (*p* value) of GO-enriched categories, and the *Y*-axis indicates the name of the item. The number of genes in the GO-enriched categories is indicated by the size of the circle.

Upregulated genes in the VB12 group were significantly enriched in pathways related to the catabolism of very long-chain and short-chain fatty acids (Figure 5), which are crucial for breaking down complex lipids into smaller, more readily metabolized molecules. The most significantly affected pathways included the very long-chain fatty acid catabolic process (*p* = 0.0074) and the short-chain fatty acid metabolic process (*p* = 0.0111). These pathways may help reduce lipid accumulation and lower the risk of lipid-related cardiovascular conditions. Furthermore, these genes were associated with steroid hormone biosynthesis, particularly in the negative regulation of steroid biosynthetic and metabolic processes, which are critical for maintaining cholesterol and hormonal balance. Key affected processes included negative regulation of the steroid biosynthetic process (*p* = 0.0192) and negative regulation of the steroid metabolic process (*p* = 0.0207). In addition, upregulated genes in the VB12 group showed enrichment in calcium-dependent cysteine-type endopeptidase activity (*p* = 0.0156), which may further contribute to regulating metabolic and inflammatory pathways. In contrast, downregulated genes were significantly enriched in pathways promoting lipid biosynthesis, such as the cholesterol biosynthetic process (*p* = 8.28 × 10^−8^), sterol biosynthetic process (*p* = 2.63 × 10^−7^), and steroid biosynthetic process (*p* = 1.84 × 10^−6^). The suppression of these pathways likely leads to a reduction in the endogenous production of cholesterol and other sterols. Additionally, positive regulators of sterol and steroid metabolism were also downregulated, which was consistent with the overall reduction in lipid biosynthesis. Lastly, the downregulated genes were enriched in pathways related to cardiac muscle hypertrophy regulation (*p* = 0.0212) and coronary heart disease (CHD)-related complexes, suggesting that VB12 supplementation may have a protective effect against pathological cardiac remodeling and CHD.

To complement DEG analysis and reveal regulatory interactions, PPI network analysis via STRING identified a connected network with 56 nodes and 83 edges (enrichment *p* < 0.001; Supplementary Figure S4) consisting entirely of downregulated DEGs post-VB12 supplementation. The network formed three main clusters: (1) a heat shock protein (HSP) and chaperone cluster (e.g., *Hsp90ab1*, *Hsp90aa1*, *Hsp90b1*, *Stip1*, *Hsph1*, *Hspb1*, *Dnajb9*, *Hspd1*, *Chordc1*, *Cacybp*, *Ern1*) involved in cellular stress response and protein folding whose downregulation may alleviate chronic inflammation and oxidative stress contributing to CVD; (2) a lipid and sterol biosynthesis cluster (e.g., *Cyp51*, *Fdps*, *Dhcr7*, *Rdh11*, *Nsdhl*, *Msmo1*) central to cholesterol and steroid metabolic pathways, with suppression aligning with reduced endogenous lipid production; and (3) a transcriptional and regulatory cluster (e.g., *Atf3*, *Myc*, *Klf10*, *Bhlhe40*, *Thrsp*, *Sall1*, *Csrnp1*, *Errfi1*, *Trim24*, *Fkbp5*, *Manf*) modulating gene expression in metabolic and developmental contexts, potentially curbing pathological remodeling. Hub genes (degree >10) primarily included a series of heat shock proteins (e.g., *Hsp90ab1*, *Hsp90aa1*, *Hsp90b1*, *Hsph1*, *Hspb1*, *Hspd1*) alongside lipid regulators (e.g., *Cyp51*, *Fdps*, *Dhcr7*, *Nsdhl*, *Msmo1*) and transcription factors (e.g., *Atf3*, *Myc*), suggesting coordinated suppression of stress and biosynthetic pathways.

## 4. Discussion

### 4.1. Assessment of Cardiovascular Risk in R. pruinosus Through Comparative Transcriptome Analysis

To determine whether *R. pruinosus* faces a similarly elevated risk of cardiovascular diseases as that observed in giant pandas and red pandas, we conducted a comparative transcriptome analysis with non-herbivorous rodents, including the Sikkim mouse, house mouse, rat, and golden hamster. Our results revealed significantly higher expression of genes involved in ether lipid metabolism, sphingolipid metabolism, and glycerophospholipid metabolism in *R. pruinosus*. Notably, *Lpcat1*, *Lpcat2*, and *Lpcat4*, which are critical for phospholipid and ether lipid synthesis, along with *Ugt8a* and *Sptlc3*, essential for sphingolipid biosynthesis, were highly expressed. The catabolism of sphingolipids involves key genes such as *Galc* and *Glb1*, while *Pla2g4c* and *Plpp3* are crucial in phospholipid degradation. Phospholipids and sphingolipids are not only vital components of cellular membranes but are also involved in neuroprotection and signal transduction, underscoring their physiological significance [58,59]. The elevated expression of these lipid metabolism genes may serve to compensate for lipid deficiencies inherent in the bamboo diet of *R. pruinosus*. In contrast, genes that were downregulated in *R. pruinosus* showed significant enrichment in pathways associated with nutrient metabolism, including fatty acid metabolism and oxidative phosphorylation, which corresponds with the intrinsically low lipid content of bamboo. Moreover, our analysis identified several genes that were significantly upregulated and closely associated with cardiovascular disease-related pathways, including those linked to dilated cardiomyopathy (DCM), hypertrophic cardiomyopathy (HCM), response to fluid shear stress, and atherosclerosis. Notable among these are *Sgcb*, *Adcy2*, *Itga1*, *Itgb8*, *Ifng*, and *Gpc1*. The aberrant expression of *Itga1* and *Itgb8* could impair vascular remodeling and endothelial integrity, thereby promoting the progression of atherosclerosis [60,61]. In addition, *Ifng* is known to exacerbate inflammatory responses in cardiovascular contexts, amplifying inflammation in arterial endothelial and smooth muscle cells and accelerating atherogenesis [62]. Collectively, these findings suggest that *R. pruinosus* may exhibit an increased vulnerability to cardiovascular diseases, potentially exceeding that of other non-herbivorous rodent species. Similarly to giant pandas and red pandas, *R. pruinosus* appears to share a heightened susceptibility to such conditions, highlighting a potential evolutionary convergence in cardiovascular risk among bamboo-feeding species. These insights emphasize the need for species-specific conservation strategies that account for unique health vulnerabilities.

### 4.2. Impact of VB12 on Cardiovascular Health in Bamboo Rats

Long-term bamboo-based diets have consistently presented cardiovascular health challenges for both giant pandas and red pandas [63,64]. Previous studies linking VB12 deficiency with adverse health outcomes have primarily focused on individuals with preexisting diseases [65,66]. Clinically, low levels of VB12 are associated with obesity, diabetes, and other metabolic disorders [67,68], all of which are established risk factors for cardiovascular diseases [69]. In our study, we used *R. pruinosus*—a species sharing dietary habits and cardiovascular risk profiles with pandas—as a model to investigate the effects of VB12 on cardiovascular health. Through transcriptomic sequencing and comparative analysis of liver samples from animals subjected to different dietary interventions, we found that exogenous VB12 intake could potentially influence cysteine and lipid metabolism. Our findings confirm that VB12 supplementation can effectively counteract key risks associated with VB12 deficiency, particularly through the reduction of Hcy levels. The significant decline in serum Hcy following VB12 supplementation directly addresses one of the primary mechanisms by which VB12 deficiency elevates cardiovascular risk. Elevated Hcy levels are well-established contributors to endothelial dysfunction and oxidative stress, both of which play important roles in the development of cardiovascular diseases. By lowering Hcy, VB12 supplementation could mitigate these risks, especially in vegetarian diets that lack natural sources of this essential micronutrient.

In addition to its effects on Hcy levels, VB12 supplementation had notable impacts on lipid metabolism. We observed significant decreases in APO-B levels, LDL levels, and the LDL/HDL ratio alongside increased APO-A levels and APO-A/APO-B ratios, indicating that VB12 improves lipid profiles favorably for cardiovascular health. In the VB12 group, we observed downregulation of genes involved in cholesterol and steroid biosynthesis, such as *CYP51A1* (encoding a lanosterol demethylase in the cholesterol pathway) and *CYP17A1* (involved in steroid synthesis), as well as *THRSP* (a regulator of lipogenesis) [70,71,72]. The reduced expression of these genes is of particular biological significance in dysregulated cholesterol metabolism, where their downregulation suppresses endogenous cholesterol production, potentially reducing atherosclerosis risk [73,74]. For instance, *CYP51A1* inhibition mimics aspects of statin therapy by limiting sterol intermediates [75], while *THRSP* downregulation curbs fatty acid synthesis in hepatic tissues [71,72]. Research involving pregnant women has shown that low VB12 levels in mothers may contribute to excess fat accumulation in their offspring, increasing the risk of type 2 diabetes and CVDs later in life [65,66].

Fatty acids are crucial components of cell membranes and key players in lipid synthesis and metabolism [76]. When fatty acid concentrations exceed physiological levels, they can cause lipotoxicity, triggering various forms of cell death and significantly elevating the risk of CVDs [77,78]. Comparative analysis between the VB12 and control groups revealed low expression of lipid synthesis genes and high expression of lipid metabolism genes in the VB12 group. In particular, key genes associated with fatty acid breakdown, regulation of steroid hormone biosynthesis, and negative regulation of lipid biosynthesis, such as *ACOT4* and *DKK3*, were expressed at significantly higher levels than in the control group. Acyl-CoA thioesterase (ACOT), located in peroxisomes, catalyzes the hydrolysis of acyl-CoA to free fatty acids and coenzyme A (CoASH), thus regulating their intracellular levels [79,80]. *ACOT* plays an essential role in lipid metabolism and is implicated in metabolic disorders. Loss of ACOT4 in mice can disrupt insulin signaling and exacerbate insulin resistance induced by a high-fat diet [81]. Additionally, *DKK3* plays a significant role in lipid metabolism by inhibiting adipocyte differentiation and glucose uptake, thus reducing adiposity and limiting body fat accumulation [82]. These findings suggest that VB12 supplementation can reduce fatty acid levels and improve lipid metabolism, potentially serving as an important factor in the prevention and treatment of CVDs.

The body contains nearly 600 proteases, which play a key role in regulating numerous physiological processes, including cysteine cathepsins (Cts), most of which are endopeptidases. While Cts predominantly function in lysosomes, extralysosomal Cts are involved in the pathogenesis of various diseases, including autoimmune disorders and cardiovascular diseases (CVDs) [83,84,85,86]. These diseases are often characterized by high levels of tissue proteases. Clinical studies have shown that tissue protease inhibitors can be used therapeutically to treat CVDs and associated complications, and inhibiting relevant pathways and gene activities may effectively mitigate cardiovascular risks [87,88]. In our transcriptomic analysis, we found two downregulated genes enriched in GO terms related to cysteine endopeptidase metabolism in the VB12 group. These genes were involved in the activation of apoptotic cysteine-type endopeptidase activity, while *Ctla2a*, a gene involved in activating cysteine endopeptidase activity, was also downregulated [89]. The decrease in homocysteine levels observed in serum biochemical analysis mirrored this trend. These results suggest that exogenous VB12 supplementation can reduce Cts expression, potentially lowering the activity of cysteine tissue proteases and thus decreasing the risk of CVDs.

Our bamboo rat model provides actionable insights for conservation biology, particularly for bamboo-dependent species like giant pandas (*Ailuropoda melanoleuca*) and red pandas (*Ailurus fulgens*) under managed care. These species face elevated CVD risks from nutrient-deficient bamboo diets, with atherosclerosis and heart failure leading causes of mortality in captivity [63,64]. Our findings—that VB12 supplementation downregulates cholesterol and sterol biosynthesis genes (e.g., *CYP51A1*, *THRSP*) and reduces serum Hcy and LDL levels—suggest targeted VB12 addition could mitigate these risks by improving lipid profiles and metabolic homeostasis, extending prior microbiome studies showing limited endogenous VB12 synthesis in pandas [90]. In managed care settings where diets are controlled, this could optimize feeding protocols, help reduce CVD incidence and enhance longevity.

While our findings provide novel insights into VB12’s role in mitigating CVD risk in bamboo specialists, several limitations must be acknowledged. Notably, the small sample (*n* = 3 per group) may reduce statistical power and limit the generalizability of subtle transcriptomic changes. This constraint stems from the challenges in sourcing larger cohorts from captive *R. pruinosus* populations, which are rare and difficult to breed due to factors like reduced litter sizes and post-pandemic declines in wildlife farming, consistent with other transcriptomic studies on specialized bamboo-feeding species, where sample sizes are often limited (e.g., *n* = 1–5) for similar ethical and logistical reasons [23]. Additionally, while we conducted qPCR validation on select upregulated genes from comparative transcriptomics (*Sgcb*, *Itga1*, *Itgb8*, and *Ifng*; consistent with RNA-seq), full experimental validation—including protein-level assessment (e.g., Western blot or immunohistochemistry) of CVD-associated downregulated genes in liver and heart tissue for the VB12 group—was not feasible due to an oversight in experimental design whereby heart tissue was not collected and the collected liver samples being fully exhausted during RNA sequencing, with *Adcy2*/*Gpc1* validation also failing due to primer design issues. Furthermore, we did not measure dietary or serum VB12 content, limiting direct confirmation that observed cardiovascular protections (e.g., Hcy reduction, lipid improvements) are attributable to VB12; however, this is indirectly supported by group-specific changes matching VB12 mechanisms [65,91]. Furthermore, while PCA revealed clear separation between the VB12 and control groups, indicating overall transcriptomic shifts due to supplementation, Spearman correlation clustering did not show strong group distinction. This discrepancy likely arises because PCA emphasizes principal sources of variance (e.g., VB12-driven changes in lipid and sterol pathways), whereas Spearman clustering, based on pairwise correlations, is more susceptible to noise or interindividual variability in small datasets, potentially masking localized effects [92,93]. Future studies should aim to validate these results with expanded samples, additional biological replicates, comprehensive functional assays including direct measurements of dietary and serum VB12 content, and multi-tissue analyses to enhance reliability, resolve such analytical inconsistencies, and explore interindividual variability.

## 5. Conclusions

This study provides preliminary evidence for the link between dietary VB12 deficiency and elevated cardiovascular disease risk in species with specialized bamboo-based diets, such as giant pandas, red pandas, and *R. pruinosus*. Through genome annotation, transcriptomic comparisons, and a controlled dietary supplementation experiment, our findings suggest that VB12 supplementation may mitigate cardiovascular risk factors in these species. The results indicate a potential role for VB12 in modulating key metabolic pathways relevant to cardiovascular health, including suppression of cholesterol synthesis, enhancement of fatty acid metabolism, and reduction in cysteine cathepsin activity. These insights offer a foundation for understanding heart disease prevention in animals with similar dietary habits, though the preliminary nature of our pilot data—limited by the small samples and partial validation—warrants further investigation in larger cohorts to confirm and extend these observations.

## Data Availability

The high-throughput sequencing data from this study have been submitted to the NCBI (project accession PRJNA1140354).

## Supplementary Materials

The following supporting information can be downloaded at https://www.mdpi.com/article/10.3390/ani15172526/s1. Figure S1: Volcano plot of differentially expressed genes (DEGs) in *R. pruinosus* vs. non-herbivorous group; Figure S2: Volcano plot of differentially expressed genes (DEGs) in VB12 group vs. control group; Figure S3: RT-qPCR validation of selected upregulated CVD-related genes from *R. pruinosus* vs. non-herbivorous rodents; Figure S4: PPI network constructed from differentially expressed genes between the VB12 group and the control group; Table S1: Information on 24 RNA-seq libraries in this study; Table S2: Primers used for RT-qPCR; Table S3: GO enrichment results for upregulated DEGs in livers of *R. pruinosus* compared to non-herbivorous rodents; Table S4: GO enrichment results for downregulated DEGs in livers of *R. pruinosus* compared to non-herbivorous rodents; Table S5: KEGG enrichment results for upregulated DEGs in livers of *R. pruinosus* compared to non-herbivorous rodents; Table S6: KEGG enrichment results for downregulated DEGs in livers of *R. pruinosus* compared to non-herbivorous rodents.
