# Supplementary material for: Impact of Vitamin B12 Supplementation on Cardiovascular Health in the Silver Star Bamboo Rat, a Species That Feeds Primarily on Bamboo
Source: Animals (Basel). 2025 Aug 27;15(17):2526. doi: 10.3390/ani15172526 (PMC12427418; doi:10.3390/ani15172526)
Supplement: Supplementary file 1 [file animals-15-02526-s001.zip › animals-3694537-supplementary/Supplementary Figures.pdf]

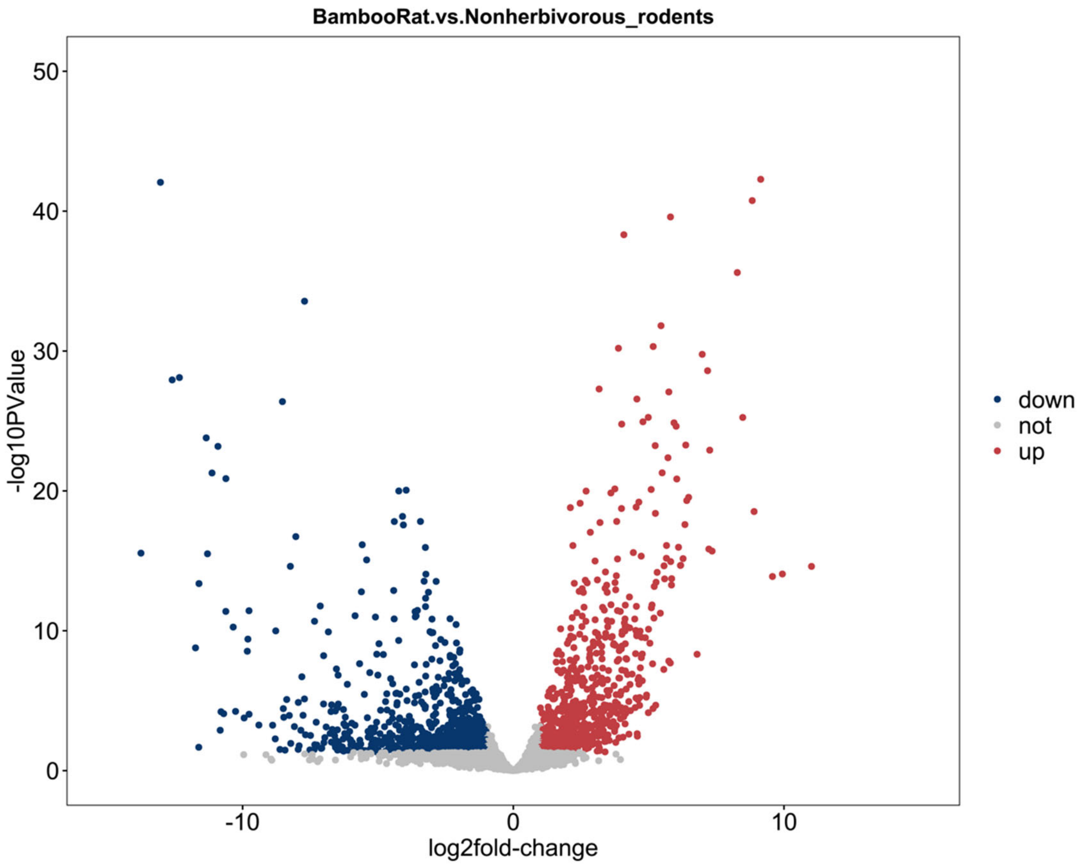


**Figure S1.** Volcano plot of differentially expressed genes (DEGs) in *R. pruinosus* vs. Non-herbivorous group. Each dot represents one gene. Red dots represent up-regulated differentially expressed genes, and blue dots represent down-regulated differentially expressed genes. Grey dots represent no significantly biased gene.

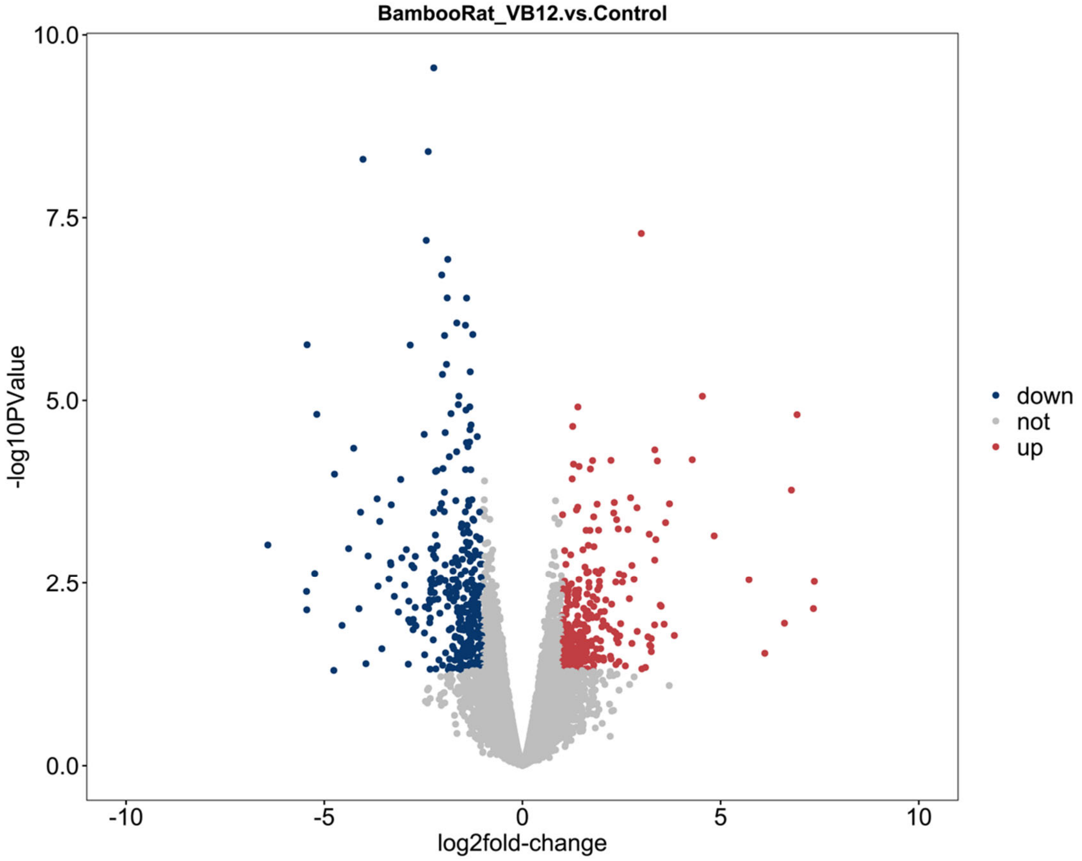


**Figure S2.** Volcano plot of differentially expressed genes (DEGs) in VB12 group vs. Control group. Each dot represents one gene. Red dots represent up-regulated differentially expressed genes, and blue dots represent down-regulated differentially expressed genes. Grey dots represent no significantly biased gene.

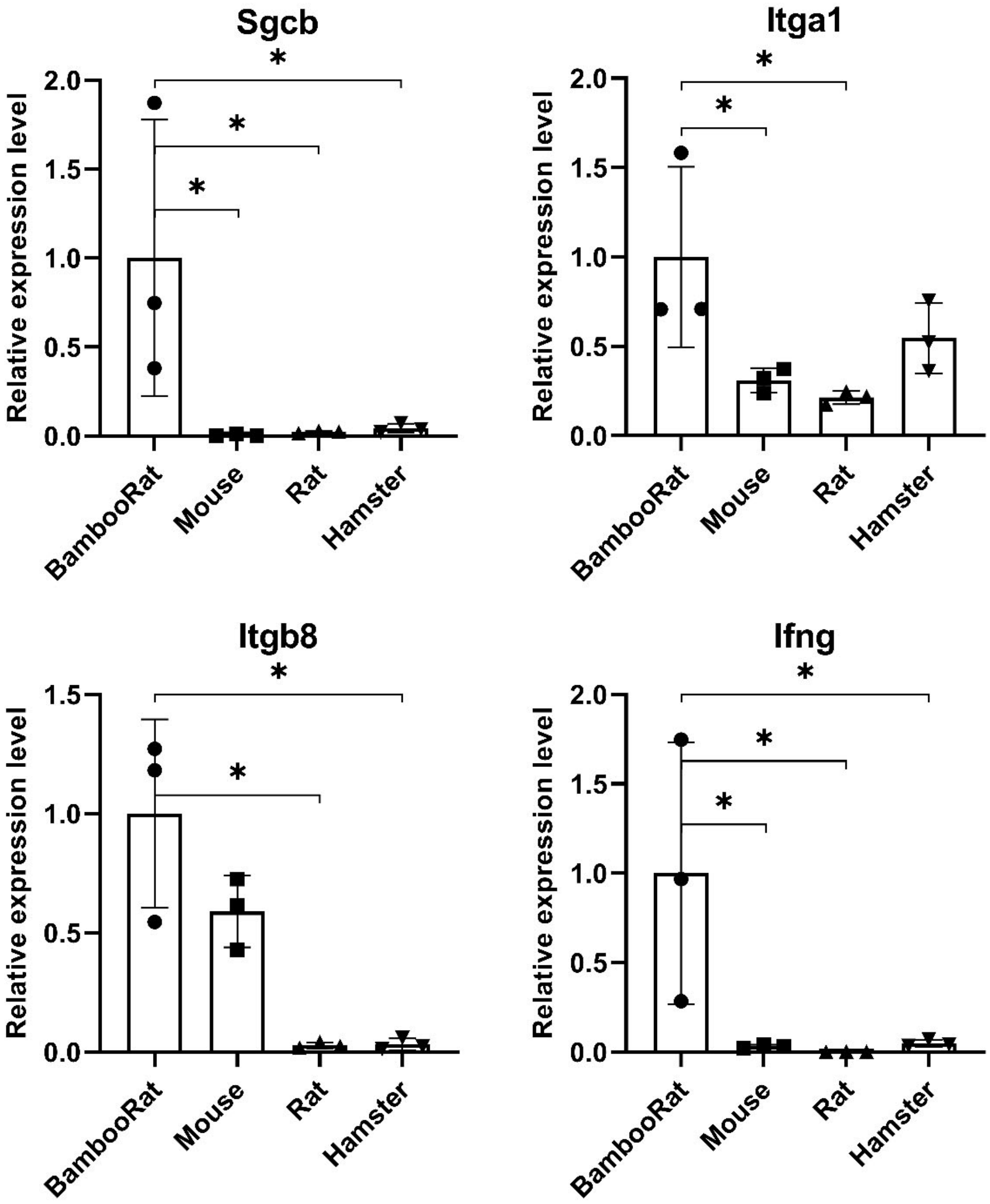


**Figure S3.** RT-qPCR validation of selected upregulated CVD-related genes from *R. pruinosus* vs. non-herbivorous rodents. The relative expression levels were calculated using the $2^{-\Delta\Delta CT}$ method, normalized to *ACTB* as the internal reference gene. Data are presented as mean ± SD ($n$=3 biological replicates per group). Statistical significance was determined by independent t-tests ($^*p < 0.05$ vs. non-herbivorous rodents). X-axis indicates different species; Y-axis represents relative expression levels.

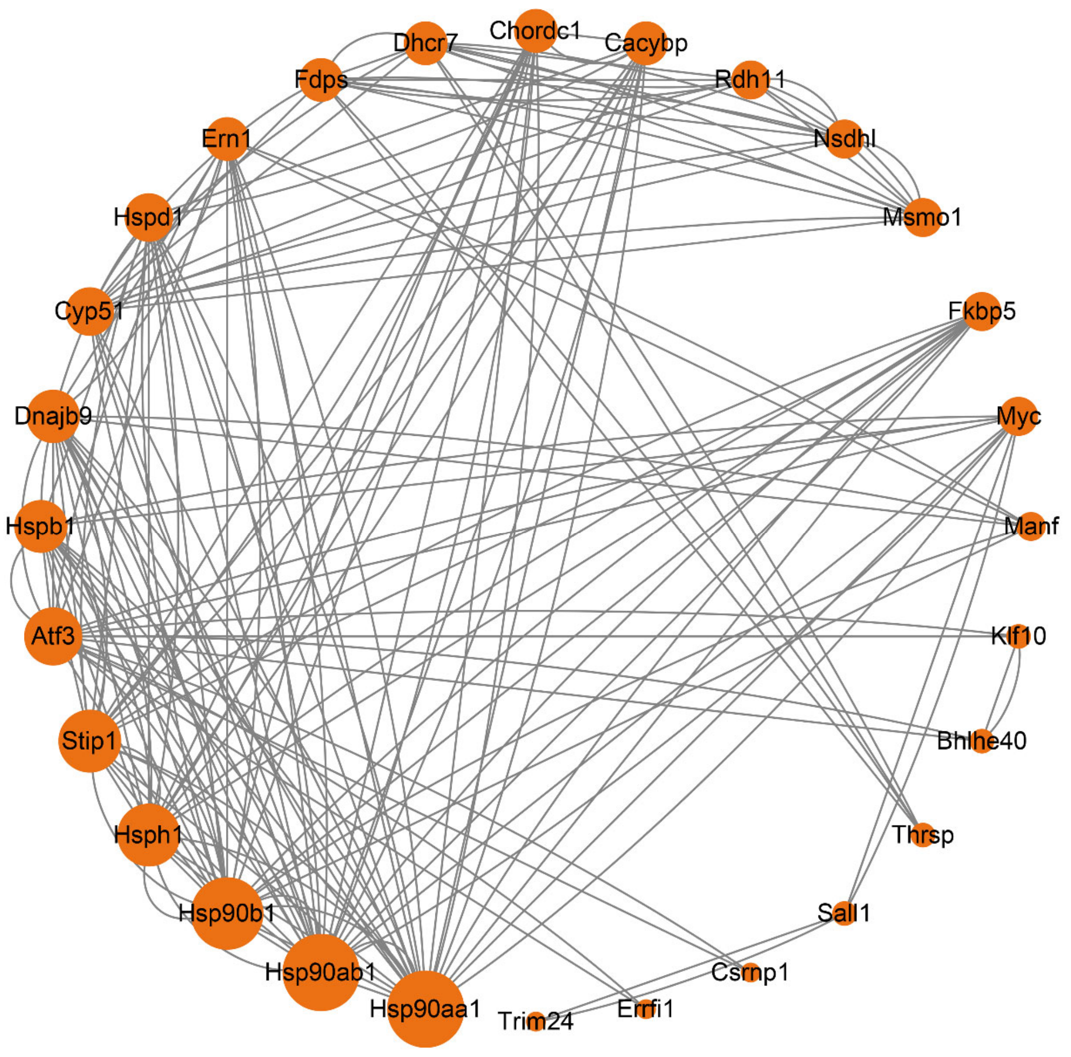


**Figure S4.** PPI network constructed from differentially expressed genes between the VB12 group and the control group. A sub-network containing hub genes is extracted. The size of the circle represents the degree of connectivity of the node gene. The larger the circle, the higher the degree of the node gene.
